# Genipin suppresses colorectal cancer cells by inhibiting the Sonic Hedgehog pathway

**DOI:** 10.18632/oncotarget.21882

**Published:** 2017-10-16

**Authors:** Bo Ram Kim, Yoon A. Jeong, Yoo Jin Na, Seong Hye Park, Min Jee Jo, Jung Lim Kim, Soyeon Jeong, Suk-Young Lee, Hong Jun Kim, Sang Cheul Oh, Dae-Hee Lee

**Affiliations:** ^1^ Department of Oncology, Korea University Guro Hospital, Seoul, Republic of Korea; ^2^ Graduate School of Medicine, Korea University College of Medicine, Seoul, Republic of Korea

**Keywords:** genipin, Hedgehog pathway, NOXA, GLI1, ubiquitin

## Abstract

Genipin, a major component of *Gardenia jasminoides* Ellis fruit, has been shown to inhibit the growth of gastric, prostate, and breast cancers. However, the anti-proliferative activity of genipin in colorectal cancer (CRC) has not been characterized. Herein, we demonstrated that genipin inhibits the proliferation of CRC cells and that genipin suppressed the Hedgehog pathway. Further investigation showed that p53 and NOXA protein levels were increased during inhibition of Hedgehog pathway-mediated apoptosis in CRC cells. We also showed that p53 modulated the expression of NOXA during genipin-induced apoptosis, and suppression via SMO also played a role in this process. Subsequently, GLI1 was ubiquitinated by the E3 ligase PCAF. In a xenograft tumor model, genipin suppressed tumor growth, which was also associated with Hedgehog inactivation. Taken together, these results suggest that genipin induces apoptosis through the Hedgehog signaling pathway by suppressing p53. These findings reveal a novel regulatory mechanism involving Hedgehog/p53/NOXA signaling in the modulation of CRC cell apoptosis and tumor-forming defects.

## INTRODUCTION

Colorectal cancer (CRC) is the third most common cancer in the world. Although the survival rate of patients with CRC has improved, it is lower than that for patient with other types of cancer [1, 2]. Furthermore, although the use of oxaliplatin and irinotecan with 5-fluorouracil has increased the overall survival rate of patients with CRC, this treatment regimen is highly toxic with high rates of side effects [3]. Therefore, alternative therapies including natural compounds have been recommended for cancer therapy [4].

**Figure 1:**
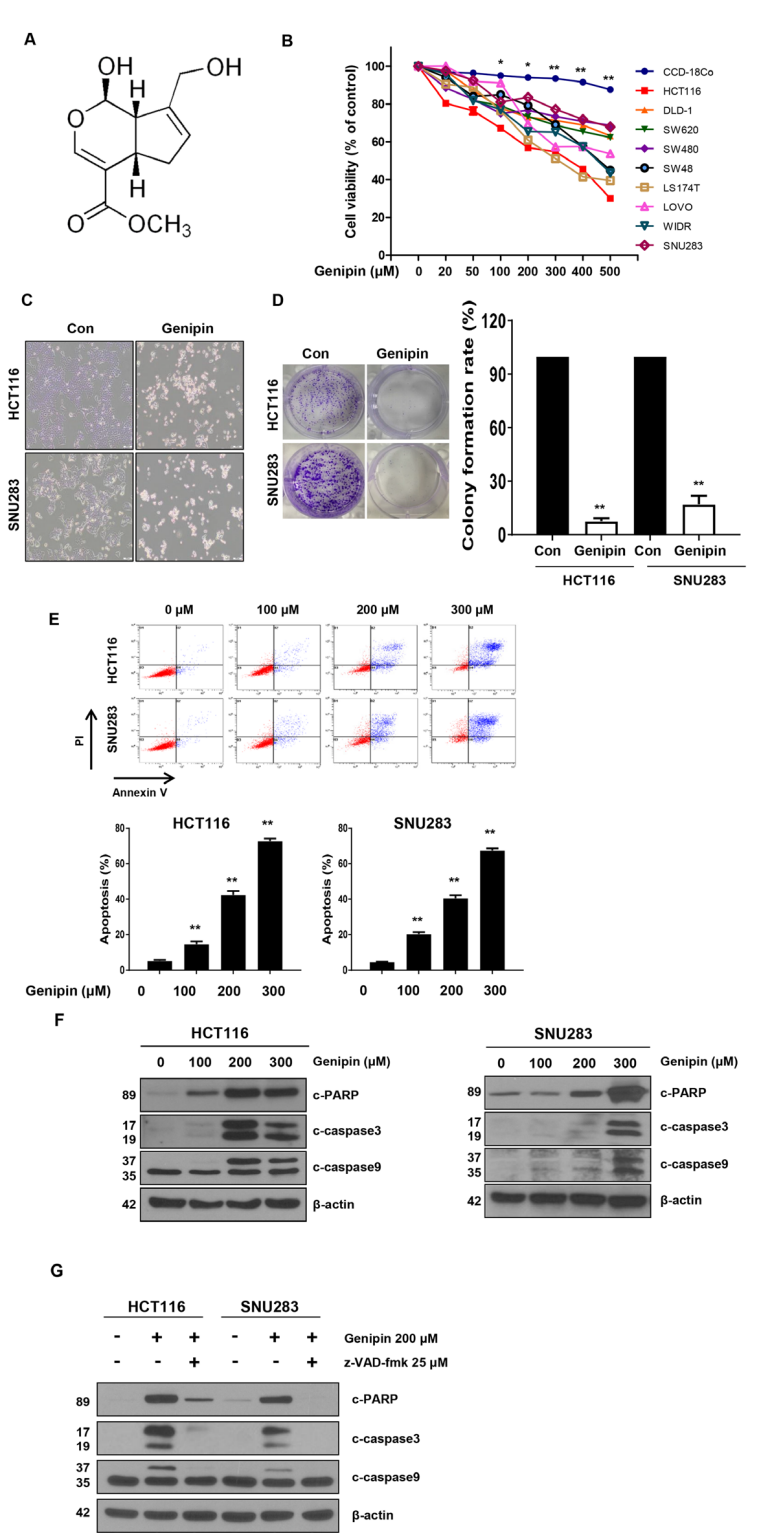
**(A)** Chemical structure of genipin. **(B)** The cell viability of colorectal cancer (CRC) cell lines measured by the MTT assay following treatment with 0–500 μM genipin for 24 h. **(C)** HCT116 and SNU283 cells were treated with 200 μM genipin, and cell morphology was examined by microscopy. **(D)** HCT116 and SNU283 cells were treated with 200 μM genipin. After 14 days, cells were stained with crystal violet and photographed (colonies shown on left). The graphs represent the percentage of stained colonies (right). **(E)** HCT116 and SNU283 cells treated with genipin were stained with annexin V and propidium iodide, and then were analyzed by FACS analysis. **(F)** The levels of cleaved PARP, CASP3, and CASP9 were detected by western blotting. β-Actin was used as a loading control. **(G)** Cells were pretreated with 25 μM z-VAD-fmk for 30 min and then treated with 200 μM genipin for 24 h. The levels of cleaved PARP, CASP3, and CASP9 were detected by western blotting. β-Actin was used as a loading control for each lane. Data are expressed as the means of three independent experiments. ^**^P < 0.01, ^*^P < 0.05.

Genipin, a major component of *Gardenia jasminoides* Ellis fruit, has effects against inflammation, ischemic brain injury, atherosclerosis, platelet aggregation, hyperglycemia, hyperlipidemia, and hypertension [5–7]. It has a molecular weight of 226 g/mol (Figure 1A), is white crystalline in structure, and exhibits low cytotoxicity. Previous studies have shown that genipin inhibits the growth of gastric, prostate, and breast cancers [8, 9]. However, the potential anti-proliferative activity of genipin in CRC has not yet been investigated.

The Hedgehog signaling pathway is closely linked to tissue polarity, patterning, and stem cell renewal during embryonic development [10]. Previous studies have demonstrated that the Hedgehog signaling pathway also plays an important role in proliferation, angiogenesis, stemness, and metastasis in various cancers [11, 12]. In vertebrates, the binding of Hedgehog ligands (Sonic, Indian, and Desert Hedgehog [SHH, IHH, and DHH, respectively]) to their receptor, patched (PTCH), results in activation of the pathway, relieving the PTCH-mediated inhibition of smoothened (SMO). Subsequently, activated SMO facilitates activation of GLI proteins, which translocate to the nucleus. Three GLI proteins, GLI1, GLI2, and GLI3, have been identified [13, 14]. GLI2 and GLI3 have C-terminal transcriptional activation domains and N-terminal repression domains, whereas GLI1 has an exclusive, full-length C-terminal transcriptional activation domain. Thus, GLI1 is the major transcriptional activator of the Hedgehog target genes. The stability of GLI1 is associated with three E3 ubiquitin ligases, the Skp/Cul/F-box complex SCF**-**β-TrCP and the E3 ligases PCAF and ITCH in conjunction with the adaptor NUMB [15–17].

The *p53* gene is the most frequently mutated gene in numerous human cancers [18]. One of the most important responses that occurs following p53 activation is the induction of apoptosis. P53 also functions to activate the transcription of several pro-apoptotic genes, such as those encoding the BH-3-only proteins NOXA, BAX, and PUMA [19]. According to previous reports, the Hedgehog signaling pathway promotes ubiquitination of p53 by MDM2, and Hedgehog signaling suppresses p53-dependent apoptosis in breast cancer [20, 21].

In the current study, we characterized the anti-proliferative activity of genipin in CRC cells. We also showed that genipin inhibited the Hedgehog signaling pathway and increased the expression of p53, which inhibited the transcriptional activity of SMO. These results suggest that genipin induces apoptosis via p53-mediated suppression of the Hedgehog signaling pathway. Therefore, inhibition of the Hedgehog signaling pathway plays an important role in cancer apoptosis.

## RESULTS

### Treatment with genipin significantly induced apoptosis in CRC cells but not in normal primary colon cells

Genipin, a constituent of *Gardenia jasminoides* Ellis fruit, is used in traditional medicine for the treatment of hepatic disorders and inflammatory diseases [5]. To investigate whether genipin can induce apoptosis in CRC cells, we treated CRC cells with genipin (0, 20, 50, 100, 200, 300, 400, and 500 μM) for 24 h. We observed that genipin induced cell death in CRC cells in a dose-dependent manner. CRC cells exhibited reduced cell viability, whereas normal colon cells (CCD-18co) were resistant to genipin (Figure 1B). As shown in Figure 1C, the morphology of the genipin-treated cells differed from that of control cells. We observed morphological changes characteristic of apoptosis, such as nuclear condensation, cell shrinkage, and blebbing, in genipin-treated cells when compared to control cells (Figure 1C). Additionally, a colony formation assay was performed to investigate the long-term effects of genipin treatment on clonogenic survival. The colony-forming ability of both the CRC and normal colon cell lines was reduced after treatment with genipin, which was consistent with the results of the apoptosis study (Figure 1D). Furthermore, apoptosis was detected by Annexin V/PI staining, and flow cytometry analysis showed that the apoptotic rates of both cell lines were increased by genipin in a dose-dependent manner (Figure 1E). To confirm these results, we evaluated the expression of pro-apoptotic markers, such as cleaved PARP, CASP9, and CASP3. The results showed that these proteins were significantly increased by genipin treatment (Figure 1F). To determine the role of caspase in genipin-induced apoptosis, we pretreated cells with z-VAD-fmk, a pan-caspase inhibitor. As expected, z-VAD-fmk significantly inhibited the genipin-induced increases in PARP, CASP9, and CASP3 (Figure 1G). These results suggested that genipin induced anti-proliferative effects in CRC cells via apoptosis.

### Genipin suppresses the Hedgehog signaling pathway

**Figure 2:**
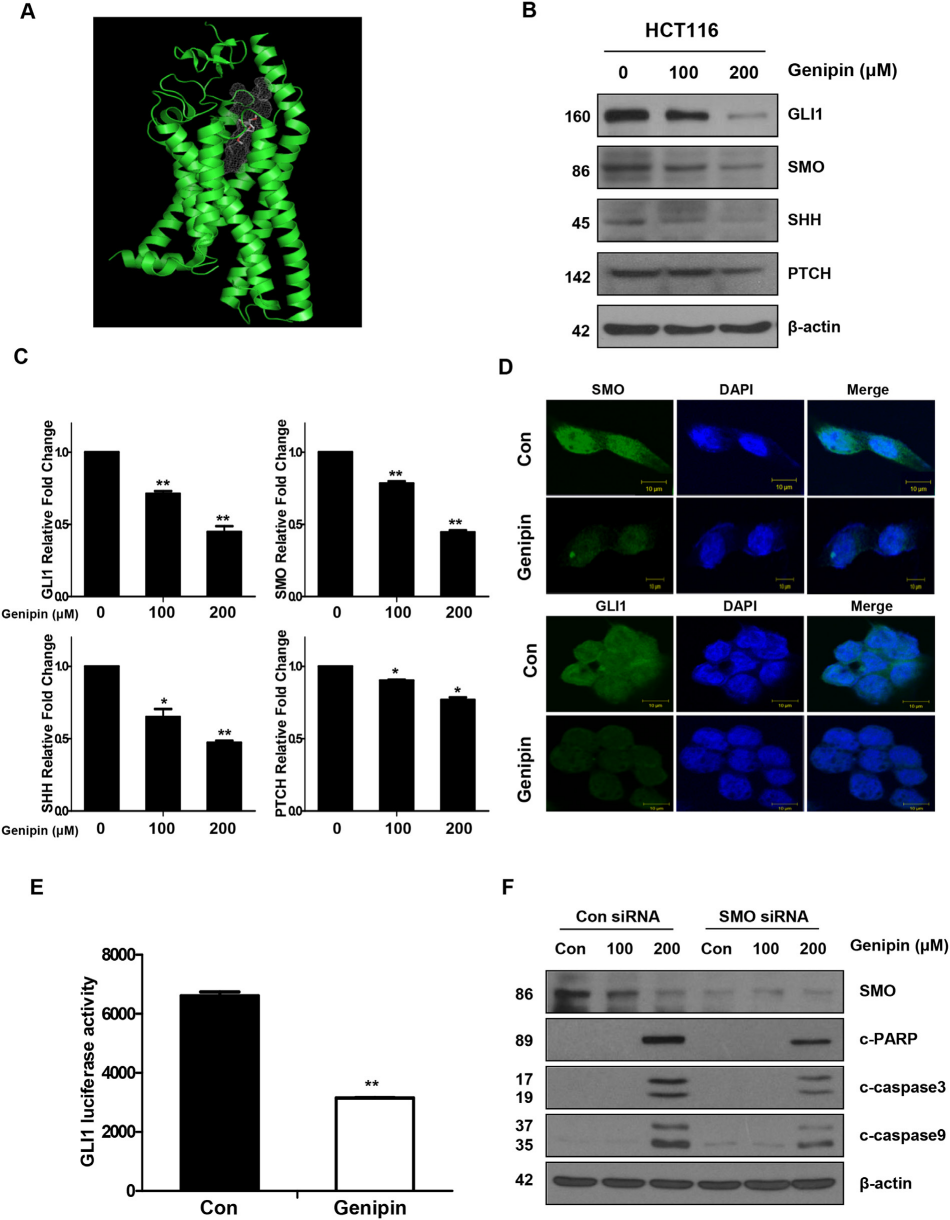
**(A)** SMO domain (green). **(B)** The protein expression levels of GLI1, SMO, SHH, and PTCH were measured by western blotting. β-Actin was used as a loading control. **(C)** The mRNA levels of *GLI1*, *SMO*, *SHH*, and *PTCH* were measured by real time PCR. Expression was normalized to that of *GAPDH*. **(D)** The immunofluorescence of SMO and GLI1 was detected by confocal laser-scanning microscopy (original magnification: 40×). Bar, 10 μm. **(E)** GLI1-luciferase activity induced by genipin. Cells were transfected with a GLI-dependent luciferase reporter construct, and then luciferase activity was normalized to that of the pRL-TK vector. **(F)** SMO was silenced in HCT116 cells with an SMO siRNA. The levels of cleaved PARP, CASP3, and CASP9 were detected by western blotting. β-Actin was used as a loading control. Data are expressed as the means of three independent experiments. ^**^P < 0.01, ^*^P < 0.05.

We showed that genipin binds to SMO (Figure 2A, Supplementary Figure 1A), and confirmed this interaction in a drug affinity responsive target stability (DARTS) assay (Supplementary Figure 1B). In DLD-1 cells, which do not express SMO, we observed no changes in cell viability, depending on genipin-mediated expression of GLI1 (Supplementary Figure 1C). However, HCT15 and HCT116 cells, which do express SMO, showed changes in cell viability, depending on the expression of GLI1 (Supplementary Figure 1D). These data indicate that genipin induced apoptosis by targeting SMO.

To determine whether genipin inhibited the Hedgehog signaling pathway in CRC cells, HCT116 cells were treated with genipin for 24 h. As shown in Figure 2B, the protein levels of SHH, SMO, and GLI1, but not PTCH, decreased in a dose-dependent manner (Figure 2B), and these results were confirmed in SNU283 cells (Supplementary Figure 2A). However, GLI2 and GLI3 levels were not changed by genipin (Supplementary Figure 2B). Consistent with the protein level results, mRNA expression analysis also showed decreases in Hedgehog signaling pathway transcripts (Figure 2C). In agreement with these observations, immunofluorescence analyses also confirmed that genipin inhibited SMO and GLI1 expression (Figure 2D). To examine the effects of genipin on GLI1, we co-transfected HCT116 cells with the 8 × 3'Gli-BS-luciferase reporter. The results showed that genipin significantly reduced GLI1 transcriptional activity (Figure 2E). Finally, we investigated whether silencing of SMO could affect apoptosis. SMO knockdown in HCT116 cells decreased genipin-induced activation of CASP3, CASP9, and PARP cleavage when compared to the levels in HCT116 control cells (Figure 2F). These results indicated that genipin induced apoptosis through inhibition of SMO.

### Genipin induces apoptosis via NOXA in p53-mediated apoptotic responses

**Figure 3:**
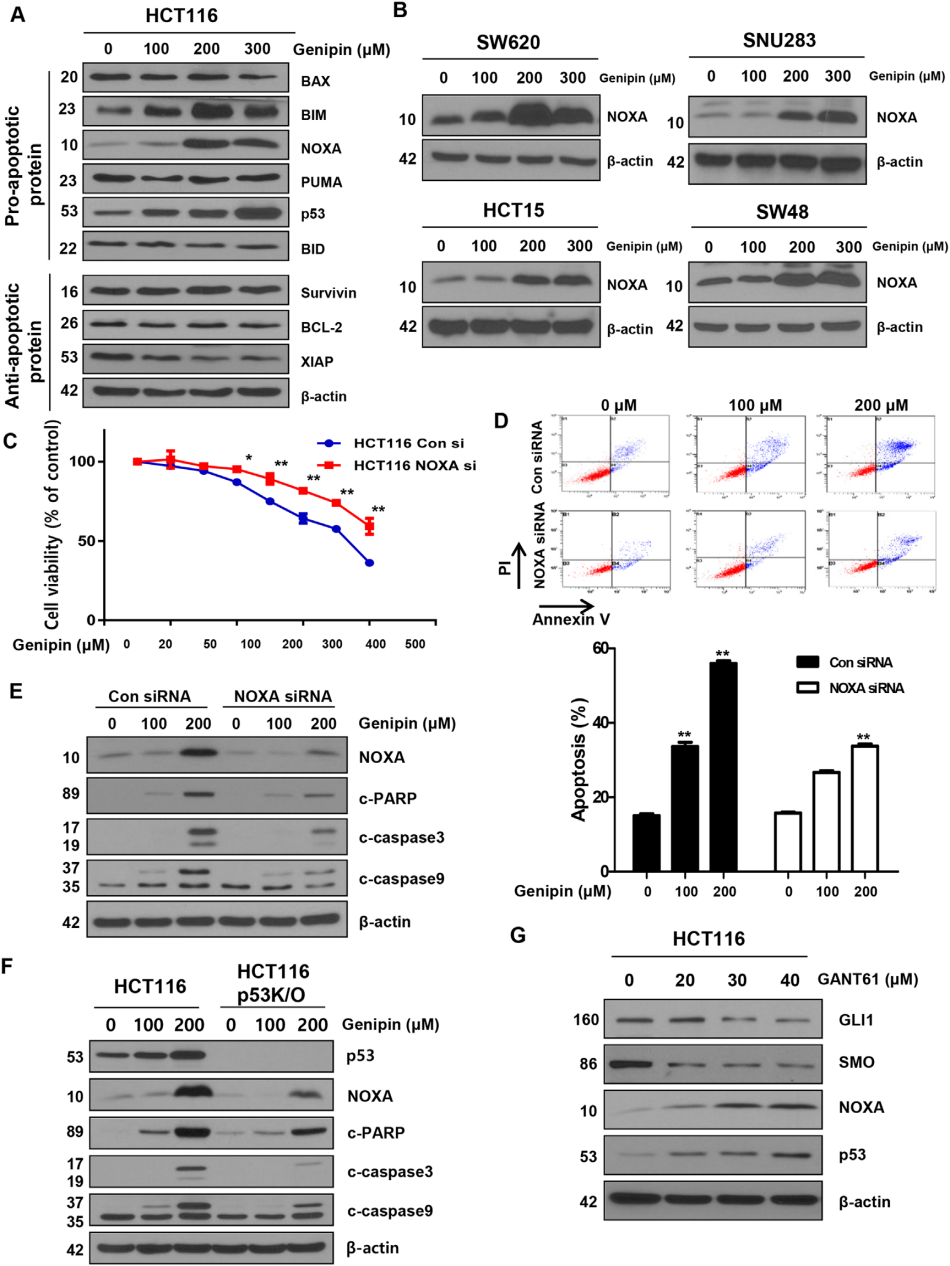
**(A)** HCT116 cells were treated with genipin (100, 200, and 300 μM) for 24 h, and then the protein levels of BAX, BIM, NOXA, PUMA, p53, BID (pro-apoptotic protein), survivin, BCL2, and XIAP (anti-apoptotic protein) were determined by western blotting. **(B)** SW620, SNU283, HCT15, and SW48 cells were treated with genipin (0, 100, 200, and 300 μM) for 24 h, and then NOXA protein levels were analyzed by western blotting. **(C)** HCT116 cells were transfected with a control or NOXA siRNA. The MTT assay was used to evaluate the effects of NOXA expression on proliferation. **(D)** HCT116 cells transfected with control or NOXA siRNA were stained with annexin V and propidium iodide (PI), and then were evaluated by using FACS analysis. **(E)** The levels of cleaved PARP, CASP3, and CASP9 were detected by western blotting. β-Actin was used as a loading control. **(F)** The levels of cleaved PARP, CASP3, and CASP9 were examined using western blotting after treatment with genipin (0, 100, and 200 μM) for 24 h. **(G)** HCT116 cells were treated with GANT61 (0, 20, 30, and 40 μM) for 48 h, and then the expression levels of GLI1, SMO, NOXA, and p53 were detected by western blotting. Data are expressed as the means of three independent experiments. ^**^P < 0.01, ^*^P < 0.05.

BCL-2 family proteins are key regulators of apoptosis. We examined the levels of caspase inhibitor protein family members, such as survivin and XIAP, and the BCL-2 family members BAX and BCL-2, and found no changes. However, in contrast, the levels of NOXA were increased by genipin in a dose-dependent manner (Figure 3A). This observation was also confirmed in SW620, SNU283, HCT15, and SW48 cells (Figure 3B). To examine whether genipin-induced apoptosis is NOXA-dependent, we silenced NOXA with a NOXA siRNA. We observed that cell viability was higher in cells treated with NOXA siRNA than in cells treated with a control siRNA (Figure 3C). As shown in Figure 3D, we also found that the apoptotic rates of HCT116 cells transfected with NOXA siRNA were significantly lower than the rates of cells transfected with the control siRNA (Figure 3D). Furthermore, NOXA knockdown decreased genipin-induced activation of CASP3, CASP9, and PARP cleavage (Figure 3E). To investigate the functional role of NOXA in the regulation of the p53 response, we examined genipin-induced apoptosis in p53-knockout (KO) cells. Compared to HCT116 cells, p53 KO cells showed decreased genipin-induced CASP3, CASP9, and PARP cleavage (Figure 3F). Finally, we investigated whether silencing of GLI1 could affect NOXA and p53 expression. Cells were treated with GANT61 (20, 30, and 40 μM) for 48 h to silence GLI1. As shown in Figure 3G, a decrease in GLI1 in 30 and 40 μM enhanced NOXA and p53 expression (Figure 3G). Taken together, these results indicate that genipin induces apoptosis via upregulation of p53-dependent NOXA.

### Genipin inhibits the Hedgehog signaling pathway by upregulating p53

**Figure 4:**
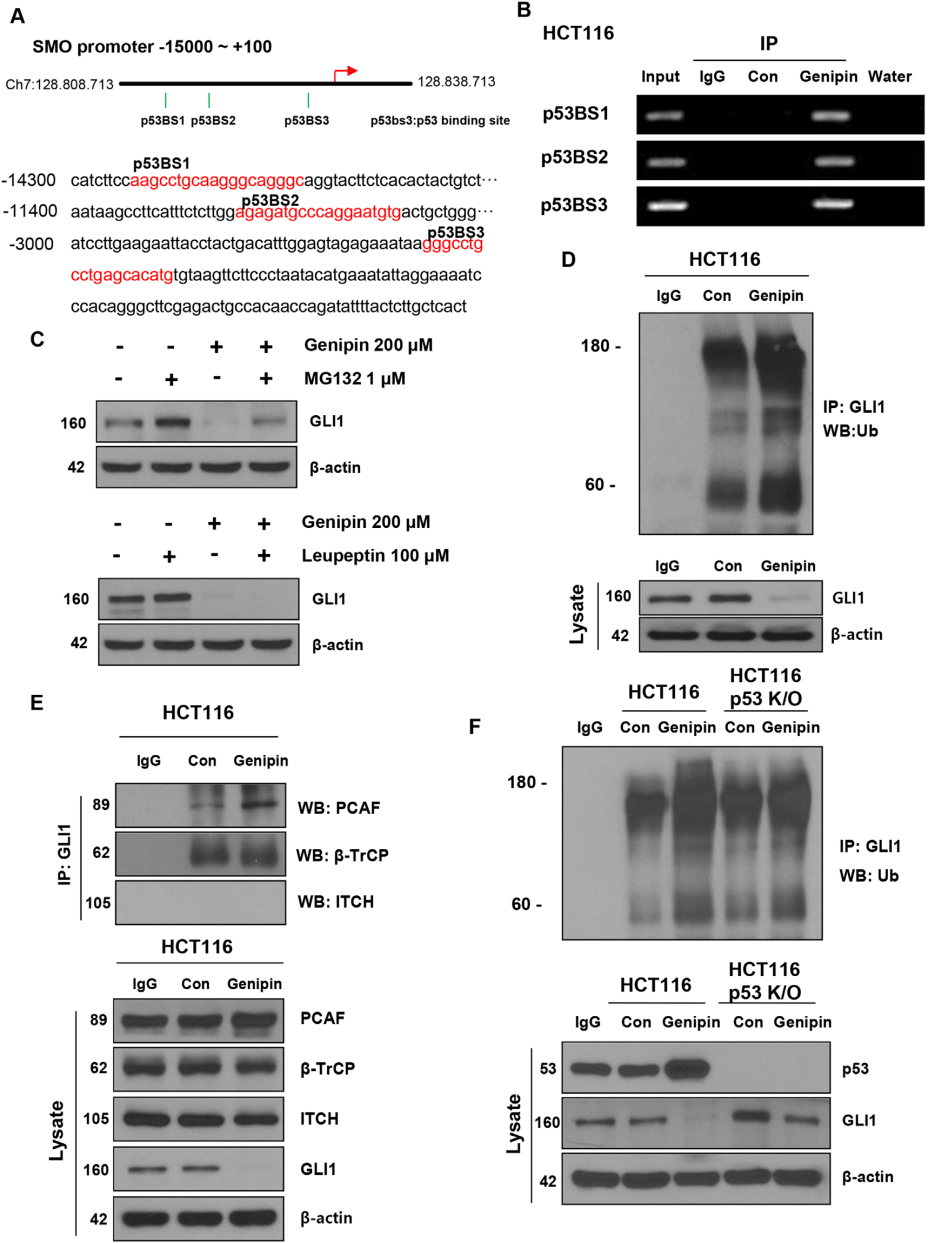
**(A)** Illustration of the three predicted p53 binding sites in the *SMO* promoter. The predicted p53 binding sites are presented in the DNA sequence of the *SMO* promoter (-15000 to -2800). **(B)** HCT116 cells were treated with 200 μM genipin, and then a chromatin immunoprecipitation (ChIP) assay was performed to confirm the direct binding of p53 to the *SMO* promoter region. **(C)** HCT116 cells were treated with 1 μM MG132 for 6 h and 100 μM Leupeptin for 24 h. GLI1 protein expression of was evaluated by western blotting. **(D)** HCT116 cell lysates were immunoprecipitated with an anti-GLI1 antibody and then immunoblotted with an anti-ubiquitin antibody. **(E)** The interaction between GLI1 and three E3 ligases was measured by co-immunoprecipitation. HCT116 cell lysates were immunoprecipitated with anti-PCAF, anti-β-TrCP, and anti-ITCH antibodies, and then immunoblotted with an anti-GLI-1 antibody. **(F)** HCT116 or p53 knockout (KO) HCT116 cells were treated with 200 μM genipin. Lysates of HCT116 and p53 KO HCT116 cells were immunoprecipitated with an anti-GLI1 antibody and immunoblotted with an anti-ubiquitin antibody. Data are expressed as the means of three independent experiments.

Since genipin affected not only SMO protein levels but also *SMO* mRNA levels, we hypothesized that genipin inhibits SMO transcription. Within the promoter region (-1–15000), we found three p53 binding sites: p53BS1 (-14292/-14274), p53BS2 (-11378/-11360), and p53BS3 (-2957/-2939; Figure 4A). We hypothesized that the genipin-induced increase in p53 may inhibit SMO expression. First, to demonstrate p53 binding to the *SMO* promoter, we performed a ChIP assay in HCT116 cells. As shown in Figure 4B, p53 binds to p53BS1, p53BS2, and p53BS3 in genipin-treated HCT116 cells. Next, to determine whether the decrease in GLI1 induced by genipin was caused by posttranslational degradation, we treated HCT116 cells with the proteasome inhibitor MG132 and the lysosome inhibitor leupeptin. When HCT116 cells were treated with 1 μM MG132 for 6 h, we observed that the genipin-induced decrease in GLI1 was blocked. In contrast, the expression of GLI1 was not changed in HCT116 cells treated with 100 μM leupeptin (Figure 4C). To investigate whether genipin could ubiquitinate GLI1, we performed an immunoprecipitation experiment. As expected, genipin induced accumulation of ubiquitinated GLI1 significant (Figure 4D). To determine whether the interactions of GLI1 with the E3 ligases β-TrCP, PCAF, and ITCH could modulate the ubiquitination of GLI1, we measured the interaction between GLI1 and E3 ligases using immunoprecipitation. Genipin increased the interaction between GLI1 and PCAF. However, the interactions between GLI1 and ITCH or β-TrCP were not affected (Figure 4E). These results showed that genipin promoted ubiquitination of GLI1 by PCAF. Additionally, to investigate whether p53 plays a key role in the ubiquitination of GLI1, we evaluated the ubiquitination of GLI1 in p53-KO HCT116 cells. As shown in Figure 4F, the increase in genipin-induced GLI1 ubiquitination was decreased in p53-KO HCT116 cells (Figure 4F); however, GLI2 and GLI3 did not induce ubiquitination (Supplementary Figure 2C). Taken together, these data suggest that p53 plays a key role in genipin-induced apoptosis.

### Genipin induces apoptosis *in vivo*

**Figure 5:**
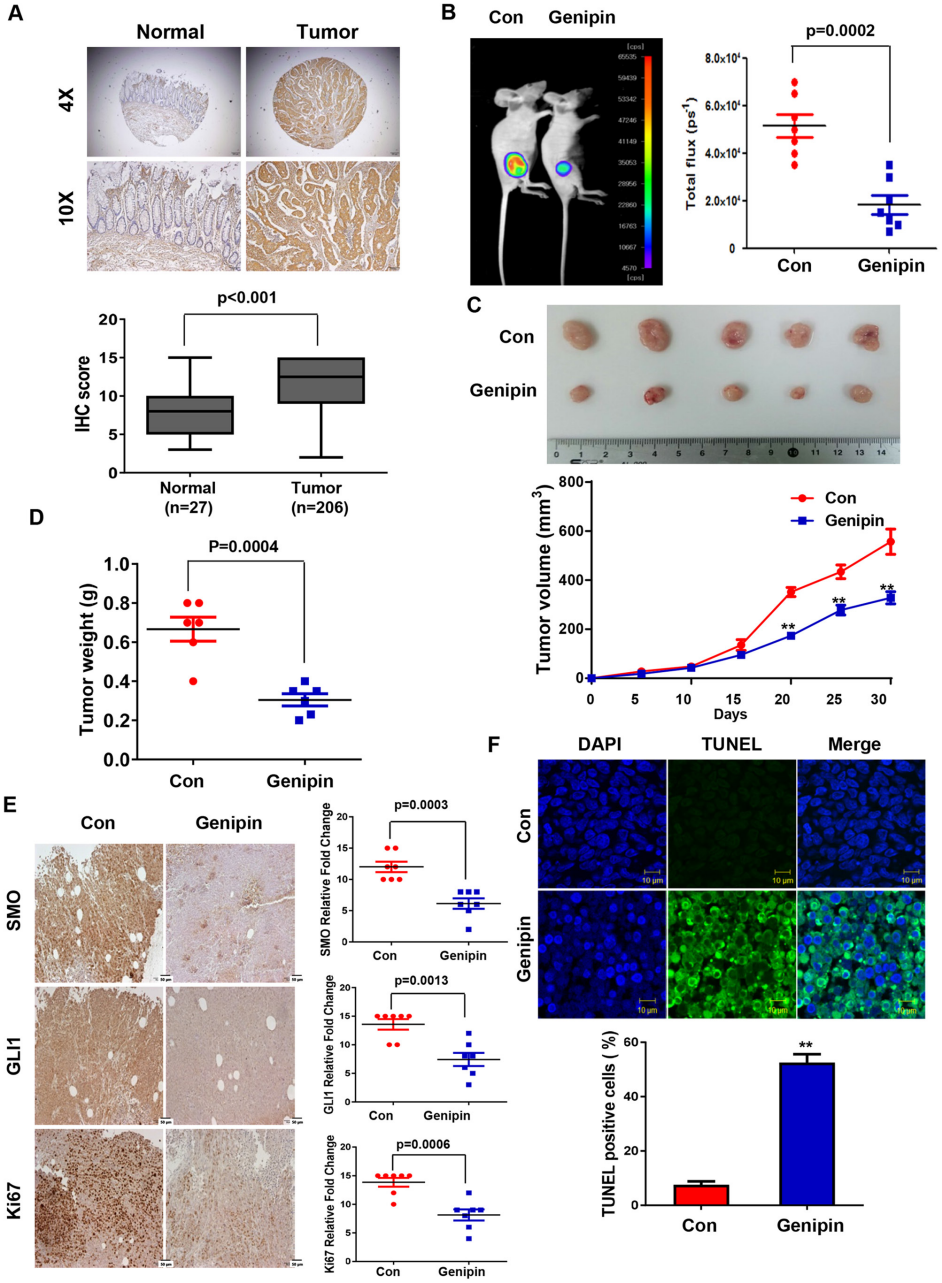
**(A)** Representative immunofluorescence image of human colon cancer specimens stained for SMO at magnifications of 4× and 10× (upper). Box plots indicate the percentage area of SMO-positive tissue in normal (n = 27) and tumor (n = 206) samples. **(B)** HCT116-luc cells were implanted subcutaneously in nude mice, and then tumor growth was evaluated by measuring fluorescent intensity after 3 weeks of treatment with genipin (20 mg/kg every 2 days; n = 8). **(C)** Representative tumor volume in xenograft nude mice. Tumor dimensions were measured every two days. **(D)** Tumor weight was measured at termination of the experiment. **(E)** Immunohistochemical (IHC) staining showing SMO, GLI1, and KI67 in tumors from xenograft mice at 20× magnification. Scale bar, 50 μm. Graphs showing IHC staining of SMO, GLI1, and KI67 (right). **(F)** Tumors were evaluated by TUNEL assay, and DAPI was used to visualize the nucleus (upper). The percentage of TUNEL-positive cells was determined and plotted as a histogram (lower).

First, IHC was used to confirm SMO expression in CRC tissues. As shown Figure 5A, SMO was highly expressed in tumors but was absent in normal colon tissue. Next, we examined whether genipin could inhibit colorectal tumorigenicity *in vivo*. For this experiment, HCT116 cells were subcutaneously injected into BALB/c nude mice. When the size of the tumors reached 100 mm^3^, the mice were randomized into two groups with and without genipin (20 mg/kg) every 2 days. Treatment with genipin significantly inhibited tumor growth compared to the tumor growth in the control group (Figure 5B and 5C). Tumor weight was lower in the genipin-treated group than in the control group (Figure 5D). Next, IHC was performed to measure the expression of SMO, GLI1, and KI67 in tumor tissue. Consistent with the *in vitro* results, treatment with genipin reduced the expression of SMO, GLI1, and KI67 (Figure 5E). Finally, a TUNEL assay was performed to evaluate apoptosis. As shown in Figure 5F, tumors treated with genipin (52.5%) had significantly more apoptotic cells than control tumors (7.5%). These results suggest that genipin induces apoptosis *in vivo*.

## DISCUSSION

Although studies have demonstrated that genipin induces apoptosis through activation of JNK or p38 [9, 22, 23], we revealed, for the first time, that genipin induces apoptosis in CRC cells. In the present study, we demonstrated that genipin induced apoptosis in human CRC cells in a dose-dependent manner via activation of p53/NOXA signaling. Our data showed that genipin inhibited proliferation and induced apoptosis in human CRC cells by inducing G0/G1 cell cycle arrest. Genipin treatment promoted inhibition of the Hedgehog signaling pathway, which triggered apoptosis, reducing in the number of CRC cells *in vitro*. Treatment of cells with the Hedgehog pathway inhibitor GANT61 or SMO siRNA augmented the cytotoxicity of genipin. We also confirmed that genipin inhibited the proliferation of human cancer cells *in vivo* via apoptosis in a human xenograft mouse model. Thus, genipin may prevent CRC, especially when combined with GANT61.

At the morphological level, apoptosis is characterized by chromatin condensation, membrane blebbing, nuclear fragmentation, and cell shrinkage. We observed these general features in genipin-treated HCT116 and SNU283 cells (Figure 1C). Furthermore, genipin-induced apoptosis, with increases in the BH-3-only protein NOXA, was enhanced in CRC cell lines (Figure 3A and 3B). NOXA interacts with anti-apoptotic BCL-2 family proteins, resulting in activation of CASP9 [24], which was activated by apoptotic signals in both p53-dependent and -independent manners. We demonstrated that genipin induced apoptosis by NOXA through a p53-dependent apoptotic response (Figure 3F).

During embryonic development, the Hedgehog signaling pathway is associated with tissue polarity, patterning, and stem cell renewal [10]. Components of the Hedgehog signaling pathway include the three hedgehog ligands, SHH, IHH, and DHH; two transmembrane protein receptors, PTCH and SMO; and three transcription factors, GLI1, GLI2, and GLI3. Previous studies have suggested that the Hedgehog signaling pathway is associated with cancer proliferation, angiogenesis, stemness, and metastasis.

The tumor suppressor p53 is a short-lived transcription factor that plays an important role in maintaining genome integrity under normal physiological conditions. Furthermore, p53 is stabilized and activated in response to a range of cellular stresses, including DNA damage, oncogene activation, hypoxia, and hyperproliferation. NOXA is a BH3-only BCL-2 family protein as well as a candidate for p53-induced apoptosis. It is likely that NOXA and other p53 target genes functionally cooperate to efficiently induce apoptosis in numerous cell types [24]. In the present study, we found that the expression levels of both p53 and NOXA were upregulated by genipin treatment in a dose-dependent manner. Pretreatment with NOXA-siRNA markedly decreased the sensitivity of CRC cells to genipin, indicating that p53 and NOXA are the major mediators of genipin-induced apoptosis. The Hedgehog signaling pathway promotes ubiquitination of p53 via MDM2, which also suppressed p53-dependent apoptosis [20]. Therefore, inhibition of the Hedgehog signaling pathway is an important strategy for cancer apoptosis. However, there are very few commercially available Hedgehog signaling inhibitors. As shown in Figure 2, genipin inhibited the Hedgehog signaling pathway, and this finding was confirmed by the results of immunofluorescence and luciferase activity assays. Because genipin affected not only SMO protein but also *SMO* mRNA, we hypothesized that genipin inhibits SMO transcription. We found that the genipin-induced increase in p53 was due to it binding to the *SMO* promoter (at p53BS1, p53BS2, and p53BS3) as shown by ChIP (Figure 4A and 4B).

GLI1 is both a transcriptional target of the Hedgehog pathway and a powerful positive activator of downstream target transcription factors. GLI1 is also regarded as a marker for activation of the Hedgehog pathway [25]. GLI1 plays a critical role in numerous types of cancer, and it promotes cancer stem cell self-renewal by inducing SNAIL expression [26]. In the present study, we found that the ubiquitin-proteasome pathway mediated regulation of GLI1 expression by genipin in CRC cells, consistent with previous findings of our laboratory and others [27–29]. Following the inhibition of SMO by p53, GLI1 was ubiquitinated by the E3 ligase PCAF (Figure 4D and 4E). However, we were unable to find the exact sequences for genipin binding to *SMO* (Supplementary Figure 1A). However, we did observed that genipin binds to *SMO* by DARTS (Supplementary Figure 1B). In addition, as shown in Supplementary Figure 1C, we found that in DLD-1 cells, which do not express SMO, genipin had no effect on cell viability, depending on the expression of GLI1 (Supplementary Figure 1C). These results indirectly suggest that genipin induced apoptosis by targeting *SMO*. Finally, we observed that GLI1 is a mediator of genipin-induced apoptotic behavior in CRC cells.

**Figure 6:**
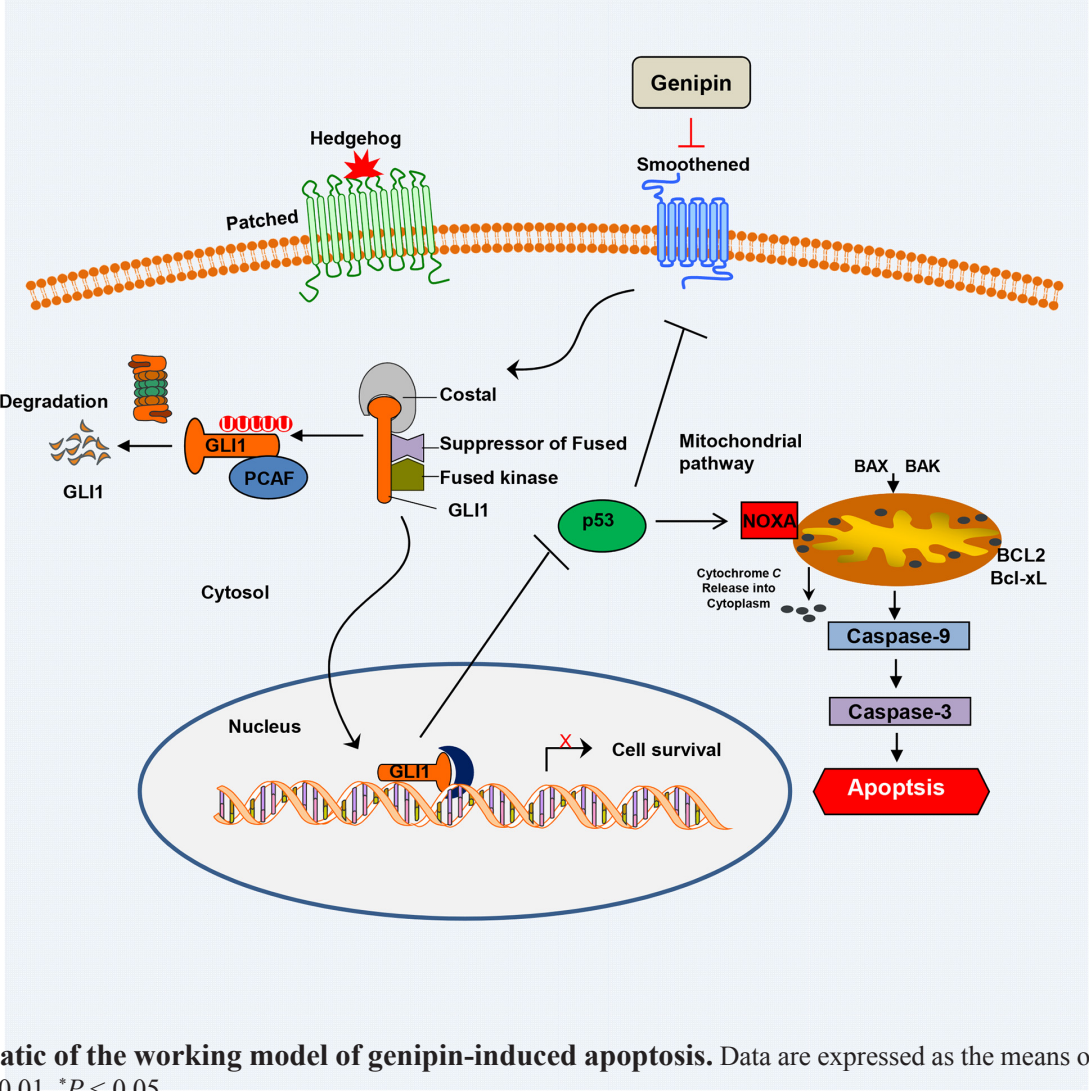
Schematic of the working model of genipin-induced apoptosis Data are expressed as the means of three independent experiments. ^**^P < 0.01, ^*^P < 0.05.

The Hedgehog signaling pathway is activated in CRC tissues [30]. Our findings support the hypothesis that inhibiting the Hedgehog pathway may be useful for treating CRC. Previous data showed that the Hedgehog pathway inhibitors GANT61 and vismodegib (GDC-0449), suppress cell proliferation and induced apoptosis [31, 32]. We combined two negative regulators of the Hedgehog pathway to treat CRC and observed synergism, suggesting that genipin in combination with GANT61 may be a good strategy for preventing CRC. Moreover, we observed significant toxicity of genipin at an intravenous dose ≥40 mg/kg, suggesting that attention must be paid to genipin-induced toxicity. Our results suggest that combinations of genipin with other anti-tumor agents, such as GANT61, may ameliorate the toxicity of genipin. Additionally, resistance to drugs, such as cetuximab, is associated with the Hedgehog signaling pathway [33, 34]. We found that genipin promotes cell death in HCT116 cells when used in combination with other anti-cancer drugs, such as cetuximab, irinotecan, and 5-FU (Supplementary Figure 3A). Therefore, inhibition of Hedgehog signaling with natural compounds, such as genipin, provides a new strategy for suppressing proliferation and/or drug resistance. To our knowledge, this is the first report of genipin-induced apoptosis, and it suggest genipin as a potential agent with potent anti-CRC activity, which may be improved when combined with other Hedgehog pathway inhibitors (Figure 6).

## MATERIALS AND METHODS

### Ethics statement

This investigation has been conducted in accordance with the ethical standards and according to the Declaration of Helsinki and according to national and international guidelines and has been approved by the authors' institutional review board. All animal procedures were carried out in accordance with the guidelines of the Korea University Institutional Animal Care and Use Committee (IACUC).

### Cell culture

Human CRC cells were obtained from American Type Culture Collection (ATCC, Manassas, VA, USA) and cultured in RPMI 1640 medium (Invitrogen, Carlsbad, CA, USA) containing 10% fetal bovine serum (HyClone, Logan, UT, USA), 1 mM L-glutamine, and 26 mM sodium bicarbonate for monolayer cell culture. The human colon cell line CCD18CO was also purchased from ATCC. All cell lines were grown in a humidified chamber at 37°C with 5% CO_2_.

### Reagents and antibodies

Genipin was purchased from Calbiochem (San Diego, CA, USA). GANT61 was purchased from Selleckchem (Houston, TX, USA). Protein G PLUS-Agarose and anti-BAX, anti-BCL2, anti-MCL-1, anti-UB, anti-BCL-Xl, anti-GLI3, anti-PCAF, anti-SHH, anti-PTCH, and anti-p53 antibodies were obtained from Santa Cruz Biotechnology (Santa Cruz, CA, USA). Anti-XIAP, anti-NOXA, anti-PUMA, anti-BIM, anti-SURVIVIN, anti-BID, anti-cleaved PARP, anti-CASP3, anti-CASP9, anti-β-TrCP, and anti-GLI1 antibodies were purchased from Cell Signaling (Beverly, MA, USA). The anti-actin antibody was obtained from Sigma (St. Louis, MO, USA), the anti-SMO antibody was obtained from Abcam (Cambridge, UK), and the anti-ITCH antibody was purchased from BD science (San Jose, CA, USA). The secondary antibodies, anti-mouse IgG HRP and anti-rabbit IgG HRP, were obtained from Cell Signaling.

### Immunoblotting

Cells were lysed in RIPA buffer (50 mM Tris, 150 mM NaCl, 1% Triton X-100, 0.1% SDS, and 1% sodium deoxycholate [pH 7.4]) containing protease and phosphatase inhibitor cocktails, and the proteins were separated by SDS-PAGE. The separated proteins were transferred to a nitrocellulose membrane (GE Healthcare Life Sciences, Pittsburgh, PA, USA), which was blocked with 5% skim milk in TBS containing 0.2% Tween 20, incubated with primary antibody, and incubated with an HRP-labeled secondary antibody. The secondary antibody signals were detected with X-ray film.

### Apoptosis assay

The translocation of phosphatidylserine, a marker of apoptosis, from the inner to outer leaflet of the plasma membrane was detected by the binding of allophycocyanin-conjugated annexin V. Briefly, untreated or genipin-treated HCT116 cells were resuspended for 24 h in the binding buffer provided in the Annexin V-FITC Apoptosis Detection Kit (Cat. LS-02-100; BioBud, SungNam, Korea). Cells were mixed with 1.25 Ml of Annexin V-FITC reagent and incubated for 30 min at 4°C in the dark. Then, staining was terminated, and the cells were immediately analyzed by flow cytometry.

### Luciferase assay

Cells were plated in 6-well plates at a density of 5 × 10^4^ cells per well and then transfected with a 8 × 3'GLI-BS luciferase construct along with the Renilla luciferase construct Pgl4 as an internal control. Cells were lysed in 100 Ml of lysis buffer (Promega, Madison, WI, USA) for 15 min at room temperature. Lysed cells were transferred into 96-well plates, and 50 μL of firefly substrate (Promega, Madison, WI, USA) was added. After measuring luciferase activity, 50 μL of Stop&Glo buffer (Promega, Madison, WI, USA) was added to the lysates. Dual luciferase activity was measured using a Glomax luminometer according to the manufacturer's instructions.

### Immunofluorescence staining

Cells grown on glass coverslips were fixed with 3.7% formaldehyde for 15 min, permeabilized with 0.5% Triton X-100 for 15 min at room temperature, and then blocked with 3% bovine serum albumin for 1 h. The cells were incubated with the primary antibodies overnight at 4°C, and then incubated with a secondary Alexa fluor-594-conjugated (Molecular Probes, Eugene, OR, USA) or FITC-conjugated secondary antibodies (Sigma-Aldrich, St. Louis, MO, USA). The nuclei were counterstained with 4',6-diamidino-2-phenylindole (DAPI) and visualized by fluorescence microscopy.

### Co-immunoprecipitation

Cells were washed with ice-cold phosphate buffered saline (PBS) and incubated with 300 μL of lysis buffer (Cat. No. 9803; Cell Signaling) containing 1 mM PMSF, protease inhibitor, and phosphatase inhibitor on ice for 5 min. Cells were harvested by scraping, and the cellular debris was removed by centrifugation at 15,000 rpm for 5 min at 4°C. Then, the protein concentration was determined by the Bicinchoninic acid (BCA) assay (Thermo Scientific, Pittsburgh, PA, USA). Cell supernatants were incubated with the primary antibody overnight at 4°C, and then 50 μL of protein G agarose beads (50% slurry) were added and incubated for 2 h at 4°C. Immunoprecipitates were washed five times with cold lysis buffer, separated by centrifugation at 10,000 rpm for 30 sec, and then mixed with 2× sample buffer and heated for electrophoresis and western blot analysis.

### Chromatin immunoprecipitation (ChIP) assay

HCT116 cells were treated with 200 μM genipin for 24 h. For ChIP, 2.5 × 10^6^ HCT116 cells were cross-linked with 1% formaldehyde at 37°C for 10 min. After washing and centrifugation, the cell pellets were lysed with SDS lysis buffer containing PMSF and protease inhibitors on ice for 10 min. The cell lysate was sonicated to shear the DNA into 200–1000 bp fragments, which were immunoprecipitated with an anti-p53 antibody at 4°C overnight. Protein-DNA complexes were collected by incubation with protein A salmon sperm DNA, eluted, and then reverse cross-linked. Following treatment with Proteinase K, EDTA, and Tris-HCl, the DNA was extracted with phenol/chloroform, precipitated with ethanol, and then analyzed by PCR using the following specific primers: p53BS1 forward, TACCTGCTTTCCTTGGTTGG and reverse, AAGGGACTTTGCAAATGGTG; p53BS2 forward, TCCGAGTTGTTGCGTGTATC and reverse, GACCCAGCAGTCACATTCCT; p53BS3 forward, AGAAATAAGGGCCTGCCTGA and reverse, GATAGCACACCCGATGCTTT.

### Animal xenograft experiment

Four-week-old female BALB/c nude mice were obtained from Orient Bio (Seongnam, Kyonggi-Do, South Korea) and housed in a specific pathogen-free environment. The animals were acclimated for 1 week prior to the study and were provided free access to food and water. HCT116 cells (1 × 10^6^) in 100 μL of culture medium were mixed with 100 μL of Matrigel and implanted subcutaneously into 5-week-old BALB/c nude female mice. Tumor size was measured every 2 days.

### Immunohistochemical staining and assessment

Sections of formalin-fixed, paraffin-embedded tumor specimens were deparaffinized in xylene and rehydrated in a graded series of ethanol. Endogenous peroxidase activity was blocked by incubation with 3% hydrogen peroxide in distilled water (D.W.) for 15 min, and antigen retrieval was performed in a cooker for 20 min. The specimens were incubated with universal blocking solution for 15 min at room temperature, and then with the primary antibodies at 4°C overnight. The antibodies, clones, and dilutions used in this assay are listed in Table 1. The sections were incubated with peroxidase-conjugated anti-goat IgG for 1 h at room temperature. Immunohistochemistry (IHC) reactions were visualized by diaminobenzidine staining using the EnVision+ system (Dako, Santa Clara, CA, United States).

**Table 1:** Antibodies used for immunohistochemical staining

Antibody	Source	Clone number	Dilution
SMO	Abcam	Polyclonal	1:100
GLI1	Santa Cruz	N-16	1:100
KI67	Santa Cruz	H-300	1:100

### Statistical analysis

Statistical analyses were carried out using GraphPad InStat 6 software (GraphPad Software, Inc., San Diego, CA, USA). The results are expressed as the mean (in arbitrary units) ± SEM. All results were evaluated using an unpaired Student's *t* test, and a *p*-value less than 0.05 was considered significant.

## SUPPLEMENTARY MATERIALS FIGURES



## Abbreviations

CRC: colorectal cancer
SMO: smoothened
GLI: Glioma-Associated Oncogene Homolog 1
PARP: poly (ADP-ribose) polymerase
PBS: phosphate-buffered saline solution
PUMA: p53 upregulated modulator of apoptosis
MDM: Mouse double minute 2 homolog
PI: propidium iodide
SDS-PAGE: sodium dodecyl sulfate polyacrylamide gel electrophoresis
PMSF: phenylmethane sulfonyl fluoride or phenylmethylsulfonyl fluoride
TBS: Tris-buffered saline
