# Correction: Genipin suppresses colorectal cancer cells by inhibiting the Sonic Hedgehog pathway

**DOI:** 10.18632/oncotarget.25677

**Published:** 2018-06-19

**Authors:** Bo Ram Kim, Yoon A. Jeong, Yoo Jin Na, Seong Hye Park, Min Jee Jo, Jung Lim Kim, Soyeon Jeong, Suk-Young Lee, Hong Jun Kim, Sang Cheul Oh, Dae-Hee Lee

**Affiliations:** ^1^ Department of Oncology, Korea University Guro Hospital, Seoul, Republic of Korea; ^2^ Graduate School of Medicine, Korea University College of Medicine, Seoul, Republic of Korea

**This article has been corrected:** The correct figure 4C is given below:

**Figure 4:**
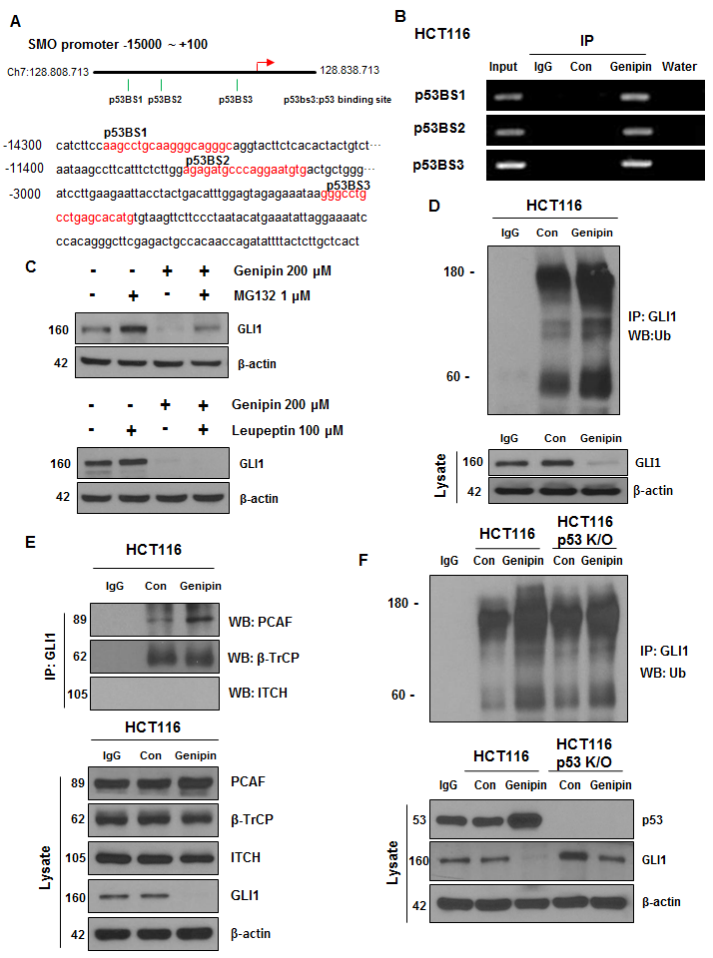
**(A)** Illustration of the three predicted p53 binding sites in the SMO promoter. The predicted p53 binding sites are presented in the DNA sequence of the *SMO* promoter (-15000 to -2800). **(B)** HCT116 cells were treated with 200 μM genipin, and then a chromatin immunoprecipitation (ChIP) assay was performed to confirm the direct binding of p53 to the SMO promoter region. **(C)** HCT116 cells were treated with 1 μM MG132 for 6 h and 100 μM Leupeptin for 24 h. GLI1 protein expression of was evaluated by western blotting. **(D)** HCT116 cell lysates were immunoprecipitated with an anti-GLI1 antibody and then immunoblotted with an anti-ubiquitin antibody. **(E)** The interaction between GLI1 and three E3 ligases was measured by co-immunoprecipitation. HCT116 cell lysates were immunoprecipitated with anti-PCAF, anti-β-TrCP, and anti-ITCH antibodies, and then immunoblotted with an anti-GLI-1 antibody. **(F)** HCT116 or p53 knockout (KO) HCT116 cells were treated with 200 μM genipin. Lysates of HCT116 and p53 KO HCT116 cells were immunoprecipitated with an anti-GLI1 antibody and immunoblotted with an anti-ubiquitin antibody. Data are expressed as the means of three independent experiments.

The authors apologize for the oversight. The authors declare that this correction does not affect the description, interpretation, or the original conclusions of the manuscript.

Original article: Oncotarget. 2017; 8:101952-101964. https://doi.org/10.18632/oncotarget.21882

