# Supplementary material for: Genipin suppresses colorectal cancer cells by inhibiting the Sonic Hedgehog pathway
Source: Oncotarget. 2017 Oct 16;8(60):101952–64. doi: 10.18632/oncotarget.21882 (PMC5731927; doi:10.18632/oncotarget.21882)
Supplement: Supplementary file 1 [file oncotarget-08-101952-s001.pdf]

Genipin suppresses colorectal cancer cells by inhibiting the Sonic Hedgehog pathway

SUPPLEMENTARY MATERIALS

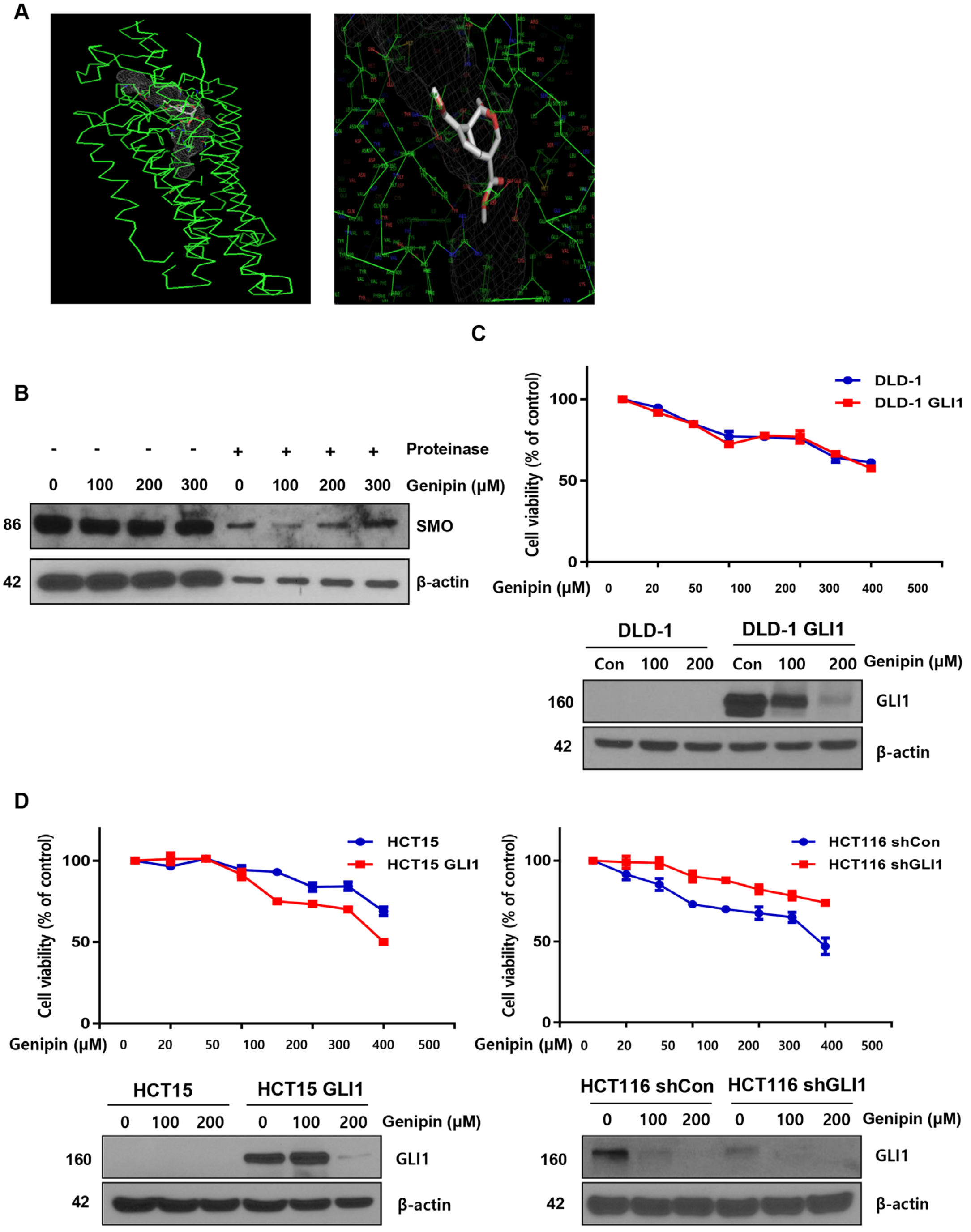

Supplementary Figure 1: (A) Binding of SMO and genipin. **(B)** Drug affinity responsive target stability (DARTS) detection by immunoblotting. SMO expression was measured, and β-actin was used as a loading control. **(C, D)** DLD-1 cells were transfected with HA-control or HA-GLI1 and the MTT assay was used to evaluate the effects of GLI1 expression on cell proliferation. Data are expressed as the means of three independent experiments.

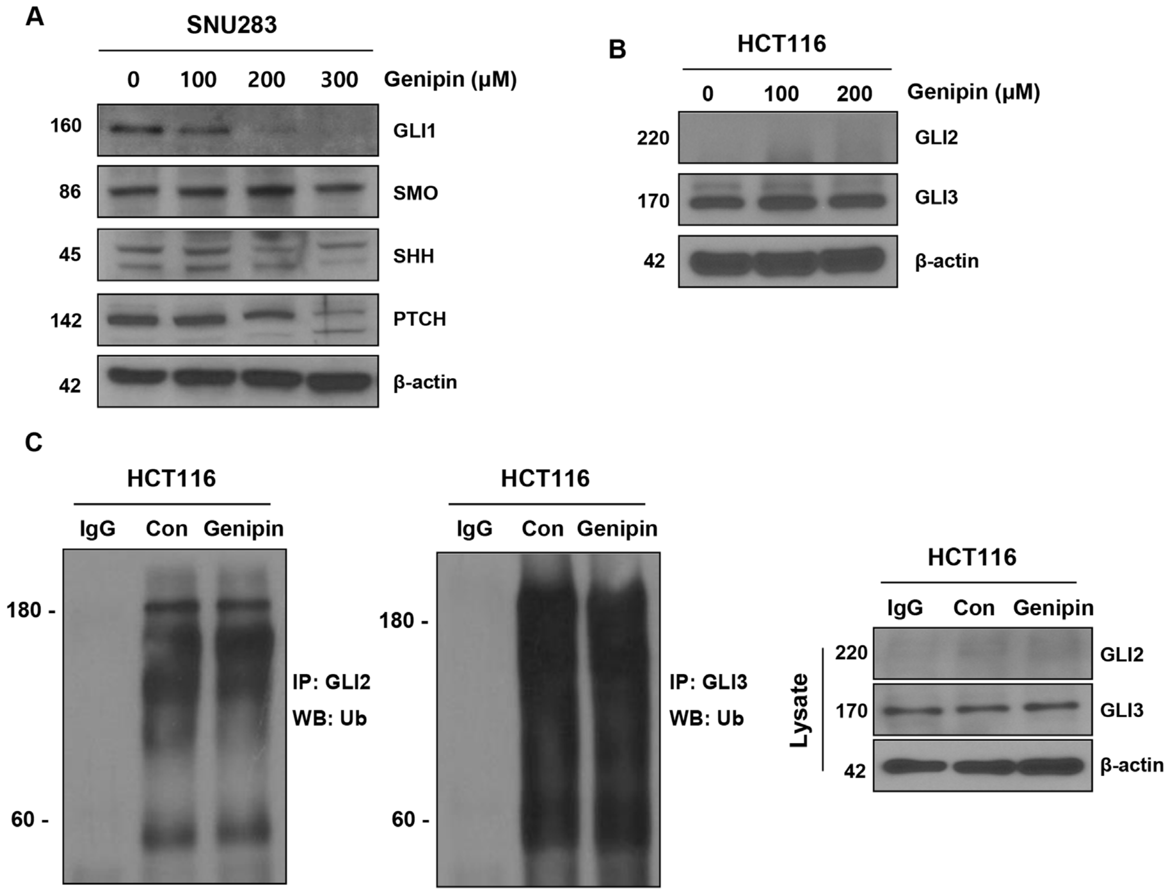

Supplementary Figure 2: (A) SNU283 cells were treated with genipin, and the expression levels of GLI1, SMO, SHH, and PTCH were measured by western blotting. β-Actin was used as a loading control. **(B)** The expression levels of GLI2 and GLI3 were measured by western blotting. **(C)** HCT116 cell lysates were immunoprecipitated with anti-GLI2 and anti-Gli-3 antibodies and then immunoblotted with an anti-ubiquitin antibody. Data are expressed as the means of three independent experiments.

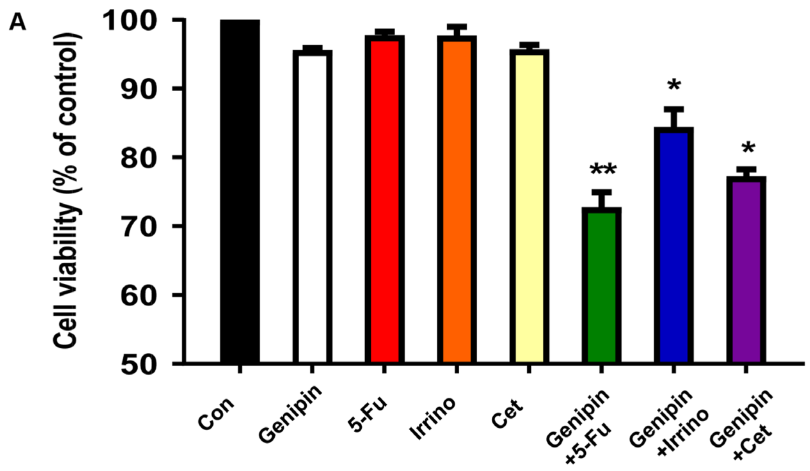

Supplementary Figure 3: (A) Susceptibility of CRCs to combined treatment with genipin and anti-cancer drugs (Cetuximab, irinotecan, and 5-FU). CRCs were treated with genipin (100 µM) and Cetuximab (10 µg/mL), irinotecan (10 µM), and 5-FU (5 µM). After 24 h of incubation, cell viability was determined by the MTT assay. Data are expressed as the means of three independent experiments.
